# Inhibitory activity of limonoids from *Khaya grandifoliola* C.DC (Meliaceae) against hepatitis C virus infection *in vitro*

**DOI:** 10.22038/AJP.2020.17215

**Published:** 2021

**Authors:** Arnaud Fondjo Kouam, Frédéric Nico Njayou, Fei Yuan, Babayemi Olawale Oladejo, He Hongtao, Pierre Mkounga, Paul Fewou Moundipa

**Affiliations:** 1 *Department of Biomedical Sciences, Faculty of Health Sciences, University of Buea, PO Box 63, Buea, Cameroon*; 2 *Laboratory of Pharmacology and Toxicology, Department of Biochemistry, Faculty of Science, University of Yaoundé 1, PO Box 812, Yaoundé, Cameroon*; 3 *CAS Key Laboratory of Pathogenic Microbiology and Immunology, Institute of Microbiology, Chinese Academy of Sciences, Beijing 100101, China*; 4 *Department of Microbiology, Federal University of Technology, P.M.B 704, Akure, Ondo State, Nigeria*; 5 *Laboratory of Physical Chemistry and Phytochemistry, Department of Organic Chemistry, University of Yaoundé 1, PO Box 812, Yaoundé, Cameroon*

**Keywords:** Khaya grandifoliola, Limonoids, Anti-HCV infection, Class III phosphatidylinositol 4-kinase alpha, 2’, 5’- oligoadenylate synthase-3

## Abstract

**Objective:**

A fraction from* Khaya grandifoliola* has recently been shown to inhibit hepatitis C virus (HCV) infection and three limonoids (17-epi-methyl-6-hydroxylangolensate, 7-deacetoxy-7-oxogedunin and 7-deacetoxy-7R-hydroxygedunin) were purified from this fraction. The present study aimed at assessing the inhibitory effect of these limonoids on HCV using cell-culture derived HCV (HCVcc) system.

**Materials and Methods:**

Cytotoxic effects of the limonoids on Huh7.5 cells were assessed by MTT assay. Huh7.5 cells were transfected with RNA transcripts of the plasmid Jc1/GLuc2a, carrying a *Gaussia* luciferase reporter gene to rescue the HCVcc particles which were used to infect naïve cells in the presence or absence of the studied limonoids during 72 hr. Infection and replication rates were monitored by luciferase reporter assay and immunofluorescence assay (IFA) while cellular gene expression was analyzed by western blot, respectively.

**Results:**

The limonoids inhibited HCV infection mostly by targeting entry and replication stage. Their inhibitory effect on entry step, comparable to that of anti-CD81 antibody, was related to the blocking of CD81 receptor. In the replication step, the limonoids decreased the expression of NS5B similar to danoprevir. These compounds also significantly decreased but up-regulated the expression of Class-III phosphatidylinositol 4-kinase alpha and 2’,5’-oligoadenylate synthase-3, respectively.

**Conclusion:**

The present findings suggest that limonoids from *K. grandifoliola *are potential anti-HCV agents and may offer an advantage in the treatment of HCV infection.

## Introduction

Hepatitis C virus (HCV) is an enveloped, positive-strand RNA virus belonging to the *Hepacivirus* genus of the *Flaviviridae* family (Moradpour et al., 2007 ▶). When HCV infects liver cells, its genomic RNA replicates and encodes a polyprotein precursor which is then cleaved by the host and viral proteases into structural proteins (i.e. the capsid core protein, two envelope glycoproteins E1 and E2, and P7 protein) and nonstructural (NS) proteins (i.e. NS2, NS3, NS4A, NS4B, NS5A and NS5B). The structural proteins form the virus particles, whereas NS proteins form the replication and assembly complex and contribute to the propagation of HCV with the help of several critical host factors (Bartenschlager et al., 2010 ▶; Bartenschlager and Sparacio, 2007 ▶; Tellinghuisen and Rice, 2002 ▶). HCV infection affects about 3% people worldwide, with a prevalence rate of 3.5% in Africa, and more than 7% for elderly people (˃45 years) in Cameroon where national rate is around 1.1% (Mohd Hanafiah et al., 2013 ▶; Njouom et al., 2015 ▶; Tietcheu Galani et al., 2016 ▶). Since its identification in 1989 (Choo et al., 1989 ▶), HCV infection has been associated with the development of liver cirrhosis, hepatocellular carcinoma, liver failure, and deaths (Ly et al., 2020 ▶).

It has been documented that binding of HCV particles to several host cell factors such as tetraspanin CD81 is highly important in the initial stage of HCV life cycle (Belouzard et al., 2011 ▶), after which, translation and replication occur in the cytoplasm. A major host cell factor regulating HCV replication that was recently identified is the Class III phosphatidylinositol 4-kinase alpha (PI4KA) (Bianco et al., 2012 ▶; Borawski et al., 2009 ▶). The assembly stage occurs in the vicinity of cytoplasmic lipid droplets and one of the host factors essential for HCV assembly is diacylglycerol acyltransferase-1 (DGAT1) which is a triglyceride-synthesizing enzyme required for core trafficking to lipid droplets (Herker et al., 2010 ▶). 

Recently, direct-acting anti-viral agents (DAAs) such as danoprevir, simeprevir, sofosbuvir and ledipasvir which are known as NS3/NS4A or NS5A/NS5B inhibitors, were developed in order to reinforce the therapeutic arsenal against HCV. Compared to the standard therapy based on the combination of pegylated-interferon with ribarivin, this new therapy possesses a 90% healing rate with fewer side effects (Keating, 2015 ▶; Liu et al., 2015 ▶). However, these new molecules are costly and not readily available in some developing countries and the standard treatments are still used. Furthermore, a limitation to the use of DAAs, is the development of resistance because of their single anti-HCV mechanism targeting only the viral factors as reported with the first DAAs, boceprevir and telaprevir, two NS3/NS4A inhibitors (Sarrazin and Zeuzem, 2010 ▶). Theses emphasize search for novel anti-HCV agents able to target both viral and host cell critical factors involved in different steps of HCV life cycle.

There is an increasing interest in the use of herbal medicines and their active constituents against viral infections which play an important role in human diseases (Lin et al., 2014 ▶). It is the case of *Khaya grandifoliola*, a medicinal plant belonging to the family Meliaceae, from which a fraction with highly promising activity against HCV was isolated (Galani et al., 2016 ▶). In addition, three known limonoids, namely 17-epi-methyl-6-hydroxylangolensate, 7-deacetoxy-7-oxogedunin and deacetoxy-7R-hydroxygedunin, were purified from the fraction and shown to protect L-02 normal human hepatocyte cell line against acetaminophen-induced hepatotoxicity (Kouam et al., 2017 ▶). However, there is no report on HCV inhibitory activity and related mechanism of action of these isolated compounds. Therefore, this work was designed to assess the inhibitory action of these limonoids against HCV infection using the cell culture-derived HCV (HCVcc) system. 

## Materials and Methods

**Chemical compounds and reagents**

Thiazolyl blue tetrazolium bromide was purchased from Sigma-Aldrich. mMESSAGE mMACHINE T7 *in vitro* transcription kit, Lipofectamine 2000 and Opti-MEM were purchased from Ambion Lifes Technologies (Carlsbad, California, USA). BioLux Gaussia Luciferase Assay Kit was purchased from NEW ENGLAND BioLabs. Renilla 5x Lysis buffer was purchased from Promega (Madison, USA). Radio-immuno-precipitation assay (RIPA) buffer was purchased from Beyotime Biotechnology (Nanjing, China); Xba1 enzyme, Halt protease inhibitor cocktail EDTA-Free 100X, Pierce bicinchoninic acid (BCA) Proteins Assay Kit, SuperSignal West Pico Chemiluminescent Substrate mouse monoclonal anti-HCV core primary antibody (1:100 dilution), secondary antibody Alexa Fluor 488 goat anti-mouse (1:1000 dilution) were all purchased from Thermo Fisher Scientific (Rockford, USA). Mouse monoclonal anti-βactin primary antibody (1:5000 dilution), horseradish peroxidase-conjugated goat anti-rabbit and anti-mouse IgG AP-linked secondary antibodies (1:2000 dilution) were purchased from Santa Cruz Biotechnology (Ca., USA). Rabbit polyclonal anti-HCV NS5B antibody (1:1000 dilution) was purchased from GeneTex (CA, USA). Rabbit polyclonal anti-DGAT-1, anti-OAS-3, anti-PI4KA (all 1:1000 dilutions) were purchased from Beijing Biosynthesis Biotechnology (Beijing, China). Danoprevir (DNV) was purchased from MedChem Express (Monmouth Junction, NJ 08852, USA). All others reagent used in this study, were of analytical grade.

**Studied compounds from** ***K. grandifoliola***

**Figure 1:**
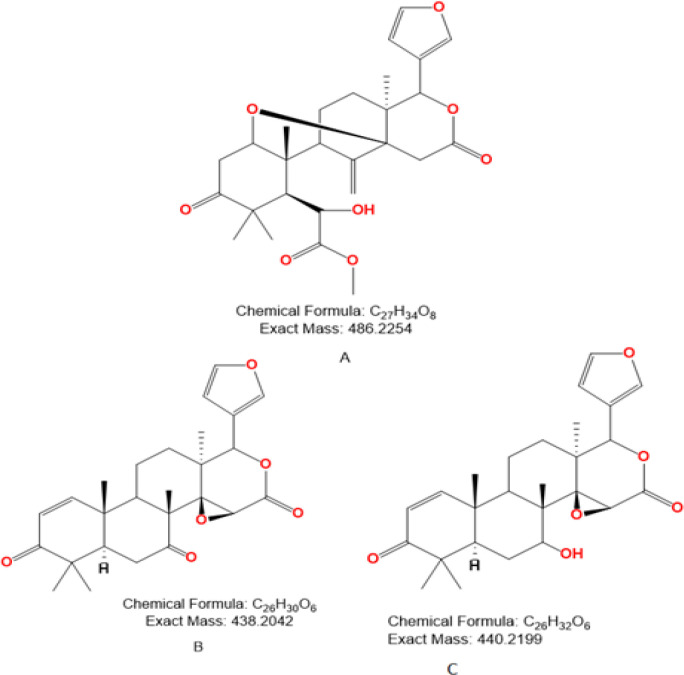
Chemical structures of studied limonoids

17-epi-methyl-6-hydroxylangolensate (C-A), 7-deacetoxy-7-oxogedunin (C-B) and deacetoxy-7R-hydroxygedunin (C-C) (Figure 1) were isolated from *K. grandifoliola* and identified using chromatography (Column, Thin Layer and High Performance Liquid Chromatography) and structural analysis (High-Resolution Mass Spectra, Nuclear Magnetic Resonance) techniques as previously described (Kouam et al., 2017 ▶). 

**Cells and culture conditions**

Highly permissive human hepatoma cells sub-line Huh7.5, (Cell Bank, Type Culture Collection of Institute of Microbiology, Chinese Academy of Sciences, Beijing, China) were cultured in 100 mm dish and maintained in high-glucose Dulbecco’s Modified Eagle’s Medium (DMEM) supplemented with 10 % fetal bovine serum in an atmosphere of 5% CO_2_ at 37°C.

**Virus strains**

The plasmid carrying the construct of Jc1/GLuc2A reporter virus (Phan et al., 2009 ▶) was generously offered by Prof. Yi Shi (Institute of Microbiology, Chinese Academy of Sciences, Beijing, China). The Jc1/GLuc2A is a derivative of the pFL-J6/JFH-1 plasmid that encodes the entire viral genome of a chimeric strain of HCV genotype 2a J6/JFH-1 (Lindenbach et al., 2005 ▶), with a luciferase gene from *Gaussia princeps* inserted between the *P7* and *NS2* genes. 

***In vitro*** **transcription**

Jc1/GLuc2A plasmid (20 µg) was linearized by Xba1 enzyme (Thermo Fischer Scientific) and purified by phenol/chloroform extraction. Purified DNA template (1 µg) was subsequently transcribed using the mMESSAGE mMACHINE T7 RNA production system (Ambion). Template DNA was removed by treatment with Turbo DNase (Ambion) at 37°C for 15 min. RNA was cleaned up by phenol/chloroform extraction and isopropanol precipitation. RNA concentration and purity were determined by reading the absorbance at 230, 260 and 280 nm using a ND-2000 NanoDrop Spectrophotometer (Thermo Scientific). 

**Transfection of Huh7.5 cells**

Twenty-four hours before transfection, Huh 7.5 cells were seeded into 6-well plate at the density of 5×10^5^ cells/well. Transfection was performed by incubating the cells with the lipofection complexes containing 5 µg of RNA transcripts and 10 µl of lipofectamine 2000 (Invitrogen) in 2 ml of serum-free medium (Opti-MEM, Invitrogen) for 12 hr. Supernatants of virus-transfected cells or control (non-transfected cells) were replaced every 3 days. Collected supernatants was clarified by centrifugation (16, 000g, 5 min, 25°C ), aliquoted and either used to measure the relative luciferase activity, or stored at -80°C as source of HCVcc in the infection study.

**Determination of 50% tissue culture infectious doses (TCID**_50_ **)**

The TCID_50_ value of viral infectivity of supernatant collected 9 days post-transfection, was determined by using an endpoint dilution assay as previously described (Lindenbach, 2009 ▶). Briefly, the virus was ten-fold serially diluted and 100 µl of each dilution was used to infect a total of 6.4×10^3^ cells/well plated onto a 96-well plate 24 hr prior to infection. Following 4 hr adsorption period, cells were washed 3 times with phosphate buffer saline (PBS) and incubated for an additional 72 hr. Supernatant was collected to measure the relative infectivity of GLuc2A and the cells were subjected to immunofluorescence assay (IFA) for HCV core protein detection.

Compound A: 17-epi-methyl-6-hydroxyangolensate; Compound B: 7-deacetoxy-7-oxogedunin and Compound C: 7-deacetoxy-7R-hydroxygedunin.

**Measurement of** ***Gaussia*** **luciferase activity**

To measure the relative infectivity of GLuc2A reporter viruses, conditioned supernatants (centrifuged at16,000×g, for 5 min at 25°C) were mixed with 0.25 volume of Renilla 5X lysis buffer (Promega, Madison) to suppress HCV infectivity. Relative lights units (RLU) were measured by a GLOMAX^TM^ 20/20 Luminometer tube reader with 20 µl sample injected and 50 µl of Gaussia luciferase solution assay reagent (New England BioLabs), integrated over 10 sec. 

**Immunofluorescence assay (IFA)**

After 72 hr of incubation, the cells were fixed using ice-cold methanol for 10 min at -20°C, blocked with 1% BSA in PBS for 30 min, and stained with anti-HCV core antibody D95B (Thermo Fisher Scientific) for 1 hr. The secondary antibody Alexa Fluor 488 goat anti-mouse (Thermo Fisher Scientific) was used to label the anti-HCV core antibody. The nucleus was stained with 1 µg/ml of 4’,6-diamino-2-phenylindole (DAPI) and imaged using a fluorescence microscope (Advanced Microscopy Groups).

**Cytotoxicity assay of studied compounds on Huh.7.5 cells**

Cells were plated in a96-well plate at the density of 6.4×10^3 ^cells/well and incubated for 24 hr. Thereafter, the medium was removed and replaced with fresh medium containing the tested compounds at various concentrations (0.1, 1, 10, 50, 100, 500 and 1000 µM) or 0.2 % dimethyl sulfoxide (DMSO) (control). After 72 hr, cell viability was measured using 3-(4, 5-dimethylthiosol-2-yl)-2, 5-diphenyl-2H-tetrazolium bromide kit (MTT; Sigma-Aldrich) according to the manufacturer’s instructions and the median lethal concentration (LC_50_) was determined.

**Anti-HCV inhibition assay**

Three sets of experiments were conducted to determine the inhibitory effect of limonoid compounds on different stages of HCV life cycle. Huh7.5 cells were seeded at the density of 6.4×10^3 ^cells/well in a96-well plate for 24 hr. The effect on the entry step was determined by inoculating Huh7.5 with the virus preparation in the presence of the limonoids for 4 hr. Then, the virus was removed and cells were incubated with fresh medium without compounds for 72 hr. To assess the effect on the replication step, cells were infected with the virus preparation in the absence of the compounds for 4 hr. Thereafter, the virus was removed and cells were incubated with fresh medium containing limonoids for 72 hr. Supernatants obtained during replication were used as extracellular source of HCVcc to infect naïve cells to study the viral assembly and release stage. After the 4-hr adsorption period, the inoculum was replaced with fresh medium without limonoids and cells were incubated for 72hr. For each step, relative infectivity was determined by measuring the GLuc2A activity as described above.

**CD81-blocking assay**

CD81-blocking assay was performed according to the method of Gottwein et al. (Gottwein et al., 2007 ▶). Briefly, Huh7.5 cells were seeded at 6.4×10^3 ^cells/well in a96-well plate. After 24 hr, cells were incubated with anti-CD81 antibody (10 µg/ml) or studied compounds at the indicated concentration for 1 hr prior infection with the virus preparation for 4 hr. Cells were washed and incubated with fresh media. After 72 hr of incubation, supernatant was collected to determine the GLuc2A activity and the cells were subjected to IFA for HCV core protein detection.

**Western blot analysis**

Huh7.5 cells were seeded at 2×10^5 ^cells/well in a12-well plate. After 24 hr, cells were infected with the virus for 4 hr. Medium was replaced with fresh media containing 0.2% DMSO, or studied compounds at the indicated concentrations. The NS3/NS4A inhibitor danoprevir (DNV) was used at 0.5 µM as positive control. After 72 hr, whole-cell extracts were prepared in radioimmunoprecipitation assay (RIPA) buffer (Beyotine Biotechnology) containing 0.2% Halt protease inhibitor cocktail EDTA-Free 100X (Thermo Scientific) and the protein concentration in each sample was quantified using Pierce BCA Proteins Assay Kit (Thermo Fisher Scientific). Thereafter, about 50 µg of total protein was subjected to SDS-PAGE and electro-transferred into a Nitrocellulose Blotting Membrane (GE Healthcare, Life Science, Germany). The membrane were blocked with 5% w/v dehydrated skimmed milk in Tris-Buffered Saline Tween-20 (TBST: 10mM Tris-HCl; 150mM NaCl; and 0.05% tween-20; pH 7.6),incubated overnight at 4°C with primary antibodies, rinsed, and then incubated for 1 hr at 25°C with horseradish peroxidase-conjugated secondary antibodies. Membrane was stained with SuperSignal West Pico Chemiluminescent Substrate (Thermo Scientific) and detection was performed by Enhanced Chemiluminescent Method which combines MicroChemi Unit and GelCapture Software. Densitometry analysis of the protein bands was performed using ImageJ Software.

**Statistical analysis**

Results are presented as mean±standard deviation (SD) of three independent experiments in triplicate. Comparisons among the mean values of various treatments groups were made by one-way analysis of variance (ANOVA) followed by the Bonferroni’s *post hoc* test whenever significant differences were observed among the variances. Differences between compared groups were considered significant at p<0.05 p<0.01 and p<0.001. Analyses were done using Prism 5.03 statistical software (Graph Pad Inc.).

## Results

**The Jc1/Gluc2A cell culture system yields high viral titers**

**Figure 2:**
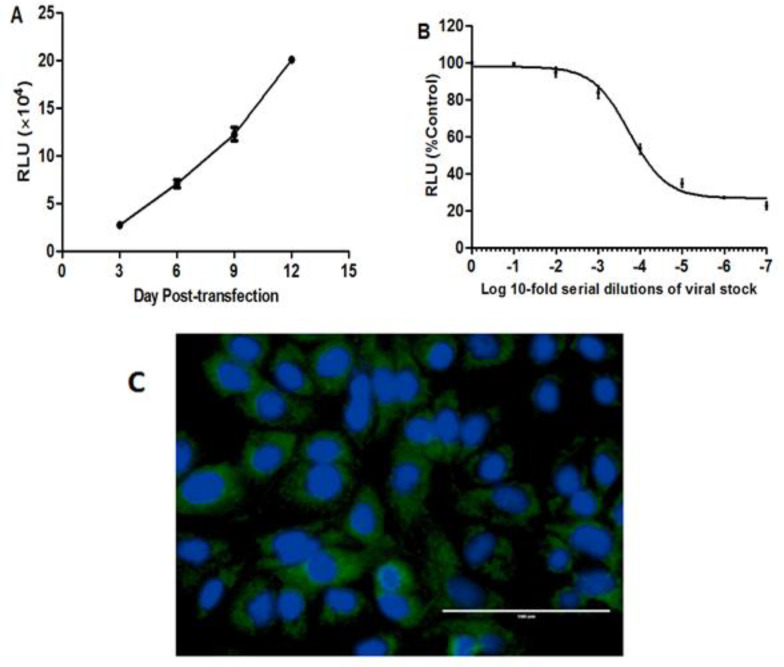
Transfection and infection of Huh7.5

The Jc1/Gluc2A reporter virus was previously engineered to secrete *Gaussia* luciferase in the cell culture medium allowing for non-invasive monitoring of viral infection and replication by measuring secreted luciferase activity without necessarily lysing the transfected or infected cells (Liu et al., 2015 ▶; Phan et al., 2009 ▶). Transfection of Jc1/Gluc2A RNA transcripts into Huh7.5 cells resulted in positive GLuc2A activity in the incubation medium after 3 days with further increase observed on the 12^th^ day (Figure 2A). The HCV infectivity titer was found to be 10^4.2^ TCID_50_/ml in the supernatant collected 9 days post-transfection serial diluted in 10-fold prior infecting naïve cells (Figure 2B). Infection of huh7.5 cells with 100 TCID_50_ resulted in HCV core immunostaining of almost all the cells after 72 hr (Figure 2C). Therefore, the viral preparation of 100 TCID_50_ was used for the anti-HCV inhibition assay.

**HCV infectivity was inhibited by limonoids at the non-toxic concentration**

**Figure 3:**
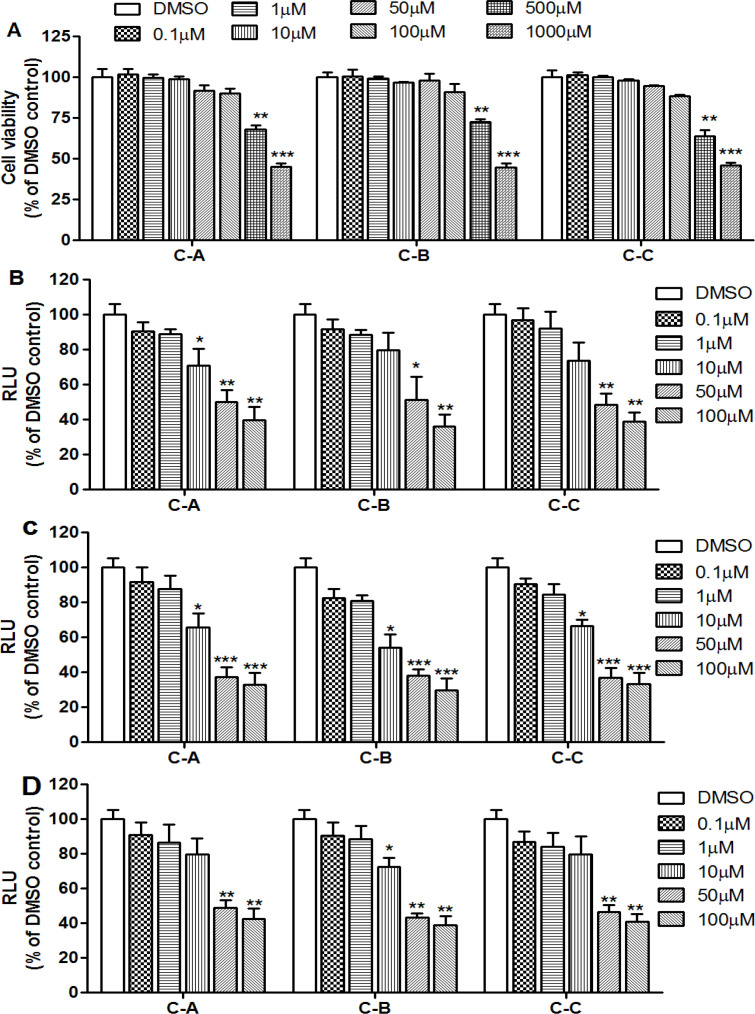
Cytotoxicity and assessment of limonoids from *K. grandifoliola* against Jc1/GLuc2A infectivity in Huh7.5 cells

Prior evaluating the antiviral properties of the limonoids, the concentration-dependent cytotoxic effect was carried out in order to determine the maximal dose which is non-toxic for the cells was analyzed by testing their effect on Huh7.5 cells at the final concentrations of 0.1, 1, 10, 50, 100, 500 and 1000 µM. The results of MTT assay showed that these limonoids exhibited similar LC_50_ values (Table 1) and up to 100 µM, cell viability in limonoids-treated cells was not significantly different from that of the control group (DMSO-treated cells) after 72 hr of incubation (Figure 3A). As such, 100 µM was therefore considered the maximal non-toxic concentration. 

Jc1/GLuc2A reporter virus was used to evaluate the effect of the limonoids on the different stages of HCV lifecycle. Huh7.5 cells were infected with 100 TCID_50_ and treated with the limonoids at concentrations up to 100 µM for each step. Relative to DMSO-treated cells, limonoid treatment at non-toxic concentration inhibited HCV infectivity during viral entry (Figure 3B), replication (Figure 3C) and release step (Figure 3D) of HCV life cycle in a dose-dependent manner. The half effective concentration (EC_50_) was determined for each step (Table 1). The therapeutic index (LC_50_/EC_50_) for C-A (17-epi-methyl-6-hydroxyangolensate), C-B (7-deacetoxy-7-oxogedunin) and C-C (7-deacetoxy-7R-hydroxygedunin) were 20.09, 17.89 and 21.17 in the entry step, 31.01, 36.45 and 31.13 in the replication step, and 24.12, 26.36 and 19.63 in the assembly/release step, respectively (Table 1). At 50 µM, the percentage of inhibition in each step was greater than 60% and this concentration was chosen for the later part of the study.

**Table 1:** Therapeutic index of limonoids tested on entry, replication and assembly steps of HCV life cycle

		**Entry**		**Replication**		**Assembly**	
	**LC**_50_ ** (µM)**	**EC**_50 _ **(µM)**	**TI**	**EC**_50 _ **(µM)**	**TI**	**EC**_50 _ **(µM)**	**TI**
C-A	877.75±13.11	41.60±4.48	21 .09	28.30±2.96	31.01	36.39±4.06	24.12
C-B	904.18±21.33	50.54±4.24	17.89	24.81±2.66	36.45	34.30±4.24	26.36
C-C	868.11±15.75	41.03±3.55	21.17	27.88±1.97	31.13	44.22±3.72	19.63

TI (LC_50_/EC_50_): Therapeutic index; LC_50_: median lethal concentration; EC_50_: half efficient concentration; C-A: 17-epi-methyl-6-hydroxyangolensate; C-B: 7-deacetoxy-7-oxogedunin; and C-C: 7-deacetoxy-7R-hydroxygedunin.

(A): Huh7.5 cells were transfected with RNA transcripts of Jc1/GLuc2A and supernatant of transfected cells was rescued every 3 days post-transfection. Harvested cell culture medium was used to assess secreted GLuc activity. (B): Supernatant collected 9 days post-transfection containing HCVcc was 10-fold serially diluted and used to infect naïve cells. GLuc activity in cell culture medium was measured after 72 hr. Results are expressed as means±SD of three independent experiments in triplicate where arrow bars represent the values of SD. (C): Huh7.5 Cells were infected with 100 TCID_50_ and subjected to immunofluorescence for HCV core protein detection after 72 hr. Image recorded usinga fluorescence microscope contains overlay of HCV core (green) and nucleus (blue).

Huh7.5 cells were treated with the indicated concentrations of limonoids and cell viability (A) was determined after 72 hr. Cells were infected with 100 TCID_50_ and treated with 0.2% DMSO or tested compounds (0.1, 1, 10, 50 and 100 µM). Gluc2A activity (Relative Light Units [RLU]) was measured in the incubation medium after 72 hr for the entry (B), replication (C) and assembly/release (D) steps of HCV life cycle. Values are means±SD of three independent experiments in triplicate; *; **; ***Values significantly different compared to DMSO control group at p˂0.05; p˂0.01 and p˂0.001, respectively as assessed by Bonferroni’s test. C-A: 17-epi-methyl-6-hydroxyangolensate; C-B: 7-deacetoxy-7-oxogedunin; and C-C: 7-deacetoxy-7R-hydroxygedunin.

**Effect of limonoids on the blocking of CD81 receptor during HCV entry**

**Figure 4:**
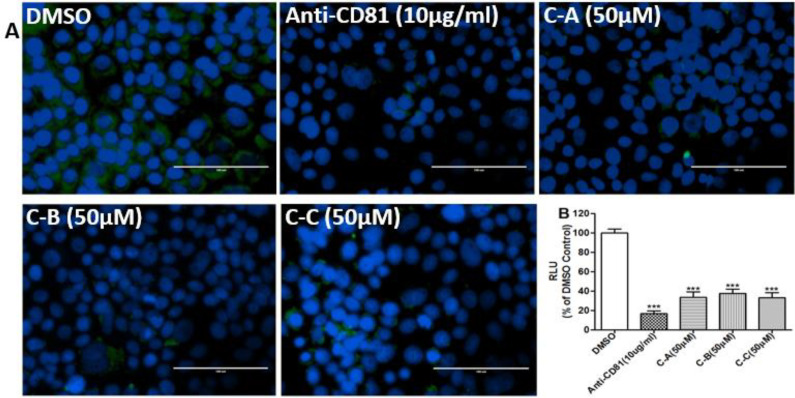
Immunofluorescence of HCV core protein detection showing inhibitory effect of limonoids from *K. grandifoliola*onHCV entry step

The entry of HCV into target cells starts from binding with host cell receptors, among which the tetraspanin CD81 receptor is mandatory (Belouzard et al., 2011 ▶; Cheng et al., 2013 ▶). It has been previously shown that HCV genotype 1a and 2a infection can be inhibited by blocking the CD81 receptor (Gottwein et al., 2007 ▶; Wakita et al., 2005 ▶). Therefore, to investigate the mechanism of the inhibitory effect of the limonoids on HCV entry, the CD81 blocking assay was performed. Pre-treatment of Huh7.5 with anti-CD81 antibody or limonoids significantly (p˂0.05) prevented infection with 100 TCID_50_ of Jc1/Gluc2A as shown by IFA for HCV core detection (Figure 4A) and GLuc2A activity (Figure 4B) compared to DMSO control. These observations in limonoids treated cells were comparable to that of anti-CD81 antibody-treated cells.

**Effect of** **l** **imonoids on the expression of NS5B, PI4KA, OAS-3 and DGAT-1**

NS5B, a RNA-dependent RNA polymerase, is a key enzyme for viral replication promoting synthesis of new RNA genomes (Kumthip and Maneekarn, 2015 ▶; Tellinghuisen and Rice, 2002 ▶). The Class-III phosphatidylinositol 4-kinase-α (PI4KA) is the major host cell factor regulating HCV replication (Bianco et al., 2012 ▶; Borawski et al., 2009 ▶) and 2’,5’- oligoadenylate synthase-3 (OAS-3) is a protein that is induced by the interferon-α signaling pathways and blocks blocked HCV replication by degrading the genomic RNA (Sen and Sarkar, 2007 ▶); the enzyme DGAT-1 is one of the major host factors mandatory for the assembly/release step of HCV life cycle (Herker et al., 2010 ▶). To gain an insight about the mechanism by which isolated limonoids interfered with HCV replication or assembly/release step, their effects on the expression of NS5B, PI4KA, OAS-3 and DGAT-1 proteins were determined. The effect of the direct-acting antiviral agent danoprevir was also examined as positive control.

Cells were pre-incubated with 0.2% DMSO, anti-CD81antibody or limonoids for 1 hr and infected with 100 TCID_50_ for 4 hr; then, cells were washed and incubated with fresh medium. After 72 hr, cells were subjected to immunofluorescence for HCV core protein detection (A). Image recorded by a fluorescence microscope contains overlay of HCV core (green) and nucleus (blue). GLuc2A activity (B) in cell culture medium was measured. Values are means±SD of three independent experiments in triplicate; ***Values significantly different compared to DMSO control group (p˂0.001) as assessed by Bonferroni’s test. C-A: 17-epi-methyl-6-hydroxyangolensate; C-B: 7-deacetoxy-7-oxogedunin; and C-C: 7-deacetoxy-7R-hydroxygedunin.

**Figure 5:**
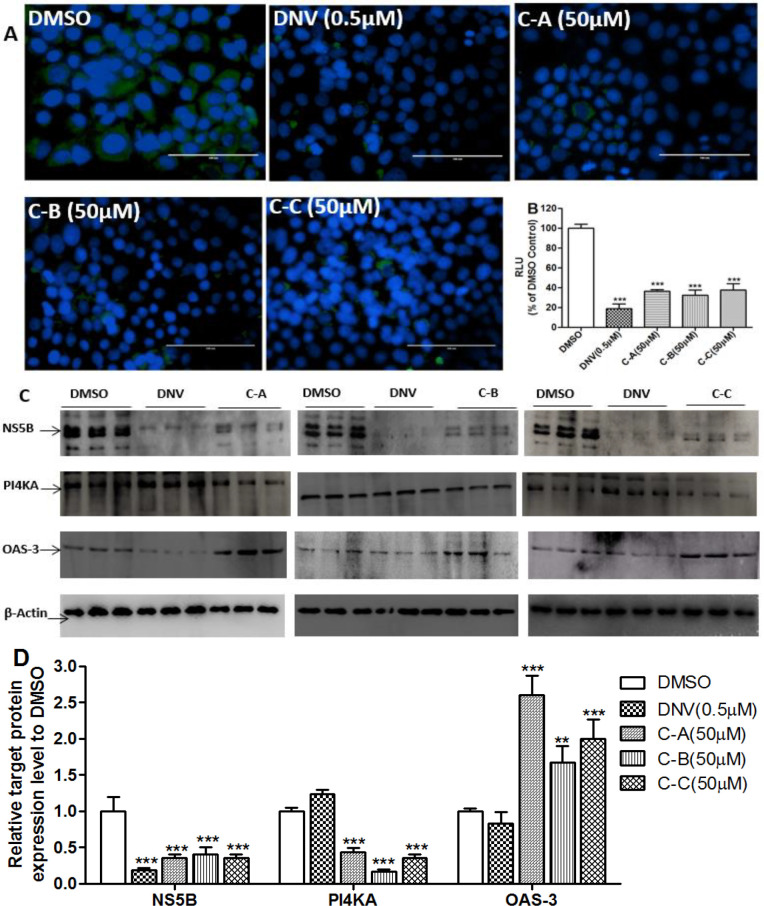
Antiviral mechanisms of tested limonoids in the replication step

**Figure 6:**
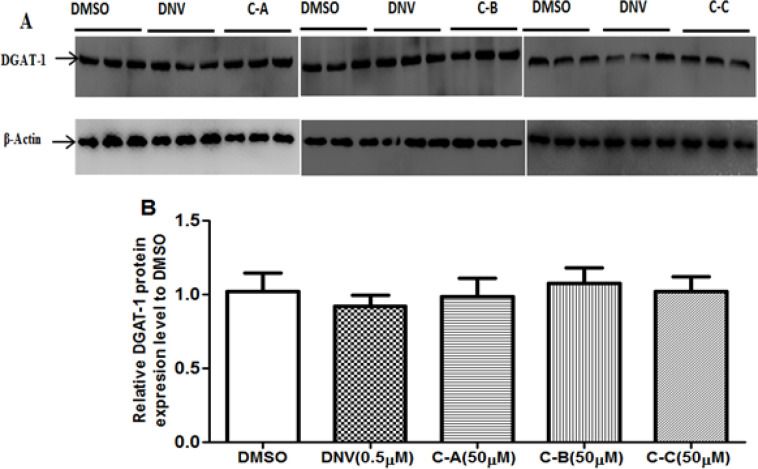
Effect of limonoids on the expression of DGAT-1

IFA (Figure 5A) and Gluc2A activity (Figure 5B) showed that the inhibitory effect of limonoids was comparable to that of danoprevir. Western blot analysis revealed that limonoids decreased the expression of NS5B in a similar way to danoprevir (Figures 5C and 5D). However, danoprevir did not affect the expression of PI4KA, or OAS-3 (Figures 5C and Figure 5D) compared to DMSO control. In contrast, in limonoids-treated cells, the expression of PI4KA was significantly (p˂0.001) reduced while the expression of OAS-3 was up-regulated up to 2.7 folds compared to DMSO control. Finally, western blot analysis showed that neither isolated limonoids nor danoprevir significantly (p˃0.05) affected the expression DGAT-1 (Figure 6). 

Cells were infected with 100 TCID_50_ for 4 hr; then,washed and incubated with fresh medium containing 0.2% DMSO, 0.5 µM DNV or 50 µM limonoids. After 72 hr, cells were subjected to immunofluorescence for HCV core protein detection (A). Image recorded bya fluorescence microscope contains overlay of HCV core (green) and nucleus (blue). GLuc2A activity (B) in cell culture medium was measured. Cells were infected with 100 TCID_50_ for 4 hr; then washed and incubated with fresh medium containing 0.2% DMSO, 0.5 µM DNV or 50 µM limonoids. After 72 hr, total proteins were extracted from cells and NS5B, PI4KA and OAS-3 expression were determined by western blotting (C). β-actin was used as loading control. (D): densitometry analysis of blots (n=3). Values are means±SD of three independent experiments in triplicate;**; ***Values significantly different compared to DMSO control group at p˂0.01 and p˂0.001, respectively; using Bonferroni’s test. C-A: 17-epi-methyl-6-hydroxyangolensate; C-B: 7-deacetoxy-7-oxogedunin; and C-C: 7-deacetoxy-7R-hydroxygedunin. DNV: danoprevir.

Huh7.5 cells were infected with 100 TCID_50_ for 4 hr; then washed and incubated with fresh medium containing 0.2% DMSO, 0.5 µM DNV or 50 µM limonoids. After 72 hr, total proteins were extracted from cells and DGAT-1 expression was determined by western blotting (A). β-actin was used as loading control. (B): densitometry analysis of blots (n=3). Values are means±SD of three independent experiments in triplicate; C-A: 17-epi-methyl-6-hydroxyangolensate; C-B: 7-deacetoxy-7-oxogedunin; and C-C: 7-deacetoxy-7R-hydroxygedunin. DNV: danoprevir

## Discussion

The inhibitory effect and the possible mechanism of action of the three compounds from *K. grandifoliola* namely 17-epi-methyl-6-hydroxylangolensate (C-A), 7-deacetoxy-7-oxogedunin (C-B) and deacetoxy-7R-hydroxygedunin (C-C), were assessed on different steps of HCV life cycle using the cell culture-derived HCV (HCVcc) system. Experimental observations showed these limonoids inhibit HCV infection mostly by targeting entry and replication steps of HCV life cycle. 

The entry of HCV into target cells starts from the interaction with several host cell receptors such as CD81, occludin, claudin-1, and scavenger receptors class-B type-1, followed by the fusion of viral and endosomal membrane (Belouzard et al., 2011 ▶). The tested limonoids inhibited the entry of HCVcc in a concentration-dependent manner (Figure 3B) with EC_50_ of 41.60±4.48, 50.54±4.24 and 41.03±3.55 µM respectively for C-A, C-B and C-C (Table 1). IFA analysis of HCV core protein (Figure 4A) and the relative luciferase activity in the incubation medium (Figure 4B) indicated that the inhibitory effect on HCV entry observed in limonoids-treated cells was comparable to that of anti-CD81 antibody-treated cells. Since blocking of CD81 receptor with specific antibodies leads to inhibition of HCV infection (Gottwein et al., 2007 ▶; Wakita et al., 2005 ▶), it can be suggested that the inhibitory effect of the limonoids on HCV entry may be related to the blocking of this receptor. Similarly, interferon-α (Kronenberger, 2001 ▶), terfenadine and salicylate (Holzer et al., 2008a ▶, 2008b ▶)were shown to inhibit HCV infection via suppression of or interaction with CD81 receptor. However, regarding the tested limonoids, their effect on the expression level of CD81 receptor remains to be investigated. 

During the viral replication process, many NS proteins and host factors are involved among which NS5B is considered to play a key role (Kumthip and Maneekarn, 2015 ▶; Liu et al., 2015 ▶) as well as the host protein PI4KA whose down-regulation inhibits HCV replication (Bianco et al., 2012 ▶; Borawski et al., 2009 ▶). Production of the cellular protein OAS-3 upon activation of IFN signaling pathways (Lehmann et al., 2010 ▶), has been shown to inhibit HCV replication by degrading the genomic RNA (Sen and Sarkar, 2007 ▶). In the present study, treatment of cells with the limonoids or danoprevir showed an inhibitory effect on HCV replication (Figures 5A and 5B). Western blot analysis (Figure 5C) revealed that the limonoids inhibited expression of NS5B in a way similar todanoprevir. In addition, compared to DMSO-treated cells, danoprevir did notaffect PI4KA or OAS-3 expression (Figure 5C) contrary to the limonoids which significantly down-regulated and up-regulated expression of PI4KA and OAS-3, respectively. These results suggest that the limonoids inhibit HCV replication, in part, through down-regulation of PI4KA. Similarly, up-regulation of OAS-3 expression by the limonoids suggests IFN signaling pathway as an antiviral target of these compounds. 

This study also showed that extracellular infectivity of HCV was significantly (p˂0.01) suppressed by the isolated limonoids (Figure 3D), indicating their inhibitory effect on HCV assembly/release step. Immunoblotting analysis (Figure 6) revealed that the limonoids did not affect the expression of DGAT-1 in this step, suggesting that this enzyme is not an anti-viral target of the tested phytochemical compounds. Thus, to further investigate this mechanism, it will be interesting to analyze the effect of these limonoids on the expression of others host factors involved in HCV assembly/release, among which the very low density lipoprotein secretion machinery (Huang et al., 2007 ▶).

This study demonstrates the inhibitory effect of limonoids isolated form *K. grandifoliola* on HCV infection *in vitro* by mainly targeting entry and replication steps of the viral cycle and may offer an advantage in the treatment of HCV infection although further studies are needed to investigate its clinical application.
